# Plasma Levels of Free Fatty Acids in Women with Gestational Diabetes and Its Intrinsic and Extrinsic Determinants: Systematic Review and Meta-Analysis

**DOI:** 10.1155/2019/7098470

**Published:** 2019-08-18

**Authors:** Jose Rafael Villafan-Bernal, Mariana Acevedo-Alba, Rodrigo Reyes-Pavon, Guillermo Andres Diaz-Parra, Diana Lucia Lip-Sosa, Hilda Imelda Vazquez-Delfin, Martha Hernandez-Muñoz, Daniel Ely Bravo-Aguirre, Francesc Figueras, Raigam Jafet Martinez-Portilla

**Affiliations:** ^1^CONACYT Cathedratic at Health Science Center, Autonomous University of Aguascalientes, Mexico; ^2^Maternal-Fetal Medicine and Therapy Research Center, Evidence-Based Health Care Department, in Behalf of the Iberoamerican Research Network in Translational, Molecular and Maternal-Fetal Medicine, Mexico City, Mexico; ^3^Mexican Consortium of Biomedicine, Biotechnology and Health Dissemination-Consortium BIO2-DIS, Mexico; ^4^Health Science Center, Autonomous University of Aguascalientes, Mexico; ^5^Fetal Medicine Research Center, BCNatal-Barcelona Center for Maternal-Fetal and Neonatal Medicine (Hospital Clínic and Hospital Sant Joan de Déu), IDIBAPS, University of Barcelona, Catalonia, Spain; ^6^Women's Hospital of the State of Aguascalientes-ISSEA, Aguascalientes, Mexico; ^7^Center for Biomedical Research on Rare Diseases (CIBER-ER), Madrid, Spain

## Abstract

**Background:**

Free fatty acids, also known as nonesterified fatty acids, are proinflammatory molecules that induce insulin resistance in nonpregnant individuals. Nevertheless, the concentration of these molecules has not been systematically addressed in pregnant women.

**Objective:**

This meta-analysis is aimed at evaluating the difference in free fatty acid plasma levels between women with gestational diabetes and healthy pregnant controls and their intrinsic and extrinsic determinants.

**Methods:**

We performed a systematic search to find relevant studies published in English and Spanish using PubMed, SCOPUS, and ISI Web of Knowledge. We included observational studies measuring the mean plasma levels of free fatty acids among gestational diabetes and healthy pregnant women, with at least ten subjects being analyzed in each group. The standardized mean difference (SMD) by random effects modeling was used. Heterogeneity was assessed using Cochran's *Q*, *H*, and *I* ^2^ statistics.

**Results:**

Among the 290 identified studies, twelve were selected for analysis. A total of 2426 women were included, from which 21% were diagnosed as having gestational diabetes. There were significantly higher levels of free fatty acids among women with gestational diabetes (SMD: 0.86; 0.54-1.18; *p* < 0.001) when compared to healthy pregnant controls and between-study heterogeneity (*I* ^2^ = 91%). The metaregression analysis showed that the gestational age at inclusion was the only cofactor influencing the mean levels of free fatty acids, indicating a trend towards lower plasma levels of free fatty acids later in gestation (estimate: -0.074; -0.143 to -0.004; *p* = 0.036). No significant publication bias was found nor a trend towards greater results in small studies.

**Conclusions:**

Women with gestational diabetes have higher levels of free fatty acids when compared to healthy pregnant controls. More investigation is needed to assess the potential role of free fatty acids in the prediction of gestational diabetes earlier in pregnancy.

## 1. Introduction

Gestational diabetes is a common disorder characterized by glucose impairment with first onset or recognition during pregnancy [1]. It is considered an important contributor to the morbidity of the mother and fetus, including hypertensive disorders, cesarean section, macrosomia, newborn hyperglycemia, shoulder dystocia, and stillbirth [1, 2].

Free fatty acids are lipids bound to albumin of mammalian blood but are also termed NEFA (nonesterified fatty acids). They are released from adipocytes after degradation of tri-, di-, and monoacylglycerols [3]. As proinflammatory molecules, free fatty acids induce insulin resistance in several organs such as skeletal muscle, pancreas, gastrointestinal organs, the liver, adipose tissue, and the hypothalamus [4], by inhibiting the tyrosine phosphorylation of IRS-1 (insulin receptor substrate-1) and reducing IRS-1-ssociated PI3K (phosphatidyl-inositol 3-kinase) activity which is responsible for transducing downstream insulin signals [5].

In nonpregnant individuals, plasma free fatty acids are increased in metabolic syndrome, mainly due to an increase in obesity as part of the diagnostic criteria [6]. In prediabetic individuals, plasma free fatty acids inhibit insulin-mediated glucose uptake, leading to further insulin resistance and type 2 diabetes [7]. Similarly, obese individuals have higher levels of plasma free fatty acids but only 50% of them will lead to failure in their compensatory mechanisms and therefore diabetes [6, 8]. Consequently, mean levels of free fatty acids are also elevated in patients with type 2 diabetes mellitus due to the same mechanisms as prediabetics and obese patients [9, 10]. Therefore, if free fatty acids are markers of insulin resistance, concentrations of these molecules should differ among women with gestational diabetes when compared to controls and even among trimesters of gestation since insulin resistance reaches its peak during the second trimester of pregnancy and decays as gestation advances [11].

The present systematic review and meta-analysis are aimed at determining whether there are any differences in the mean plasma levels of free fatty acids among women with gestational diabetes compared to healthy pregnant controls and the influence of gestational age at diagnosis, pregestational body mass index (BMI), fasting glucose, fasting insulin, mean maternal age, and year of publication of the studies, on the pooled results.

## 2. Methods

### 2.1. Protocol Registration

Before running the systematic search and data extraction, the protocol was agreed between authors and published in the PROSPERO international prospective register of systematic reviews (registration number: CRD42019124648).

### 2.2. Eligibility Criteria, Information Sources, and Search Strategy

A systematic literature search was made using PubMed, ISI Web of Science, and SCOPUS, to identify relevant studies published in English and Spanish, without time limit. The following keywords were used: (“fatty acids, nonesterified”[MeSH Terms] OR (“fatty”[All Fields] AND “acids”[All Fields] AND “nonesterified”[All Fields]) OR “nonesterified fatty acids”[All Fields] OR (“free”[All Fields] AND “fatty”[All Fields] AND “acids”[All Fields]) OR “free fatty acids”[All Fields]) AND (“diabetes, gestational”[MeSH Terms] OR (“diabetes”[All Fields] AND “gestational”[All Fields]) OR “gestational diabetes”[All Fields] OR (“gestational”[All Fields] AND “diabetes”[All Fields])) AND “humans”[MeSH Terms]. A manual search was also used for additional potentially relevant studies. The first search was run on December 15, 2017, and updated on March 5, 2019.

This systematic review was conducted adhering to the meta-analysis of observational studies in epidemiology (MOOSE) guidelines [12] and the PRISMA (Preferred Reporting Items for Systematic Reviews and Meta-Analyses) guidelines for systematic reviews and meta-analysis [13], as previously performed in similar meta-analysis [14]. Two independent investigators evaluated the identified abstracts (J.R.V.B. and M.A.A.), both blinded to authorship, authors' institutional affiliation, and study results. In case of any disagreement, a third investigator (R.J.M.P.) resolved it. After the selection of abstracts that fulfilled the inclusion criteria, a second full-text revision was made. For relevant studies with missing information, corresponding or first authors were contacted by e-mail to request the data. [Supplementary-material supplementary-material-1] of the supplemental material details the search strategy and query syntaxes.

### 2.3. Study Selection Criteria

The following inclusion criteria were used for article selection: observational studies measuring the mean levels of free fatty acids among pregnant women with and without gestational diabetes mellitus during the second and third trimesters. We decided to exclude articles with no reported control group, studies measuring free fatty acids in the first trimester only, and studies with less than five patients in any of the included groups. Reasons for excluding articles measuring free fatty acids in the first trimester are due to the high probability that first trimester cases are the result of a previous nondiagnosed type 2 diabetes rather than normal pregnancy with gestational diabetes alone.

### 2.4. Data Extraction and Quality Assessment

The following information was derived on a datasheet based on Cochrane Consumers and Communication Review Group's data extraction template: author, year of publication, country where the study was conducted, study methodology, exclusion and inclusion criteria, overall included patients, number of participants with gestational diabetes, number of nondiabetic pregnant women, free fatty acid fasting plasma levels, fatty acid quantification method, mean maternal age at analysis, mean pregestational maternal body mass index (pBMI), mean gestational age at measurement of free fatty acids, fasting plasma glucose, fasting plasma insulin, and fasting glycated hemoglobin (HbA1c).

### 2.5. Assessment of Risk of Bias

Two reviewers (R.J.M.P. and R.R.P.) independently evaluated the quality of the selected articles. The quality assessment of the observational studies was carried out using the Newcastle-Ottawa scale for case-control studies. Each article was evaluated on three main dimensions: selection of the study groups, the ascertainment of the exposure, and the comparability of the groups. A star was given for each signaling question among each dimension. For a total of nine possible stars, studies with seven or more stars were considered as high quality [15].

### 2.6. Statistical Analysis

Extracted results were pooled in the meta-analysis. Data analysis was performed in the following manner: mean levels of free fatty acids within the comparison of gestational diabetes and nondiabetic pregnant controls. The effect size was expressed as the standardized mean difference (SMD) by random effects model [16] defined as the mean difference in mean outcome between groups divided by the standard deviation of outcome among participants [17]. Results were presented using Forest plots. Between-study variability was assessed using the *τ* ^2^, Cochran's *Q*, and *I* ^2^ statistics [18]. A subgroup analysis was performed to evaluate the SMD of free fatty acid plasma levels according to the trimester of gestation. Multiple metaregressions were also performed to assess the influence of several covariates on the pooled SMD. The following covariates were used for the metaregression: mean gestational age at analysis, mean maternal age, body mass index, mean plasma levels of fasting glucose, mean plasma levels of fasting insulin, and year of publication. Publication bias was assessed by Egger method and plotted as contour-enhanced funnel plots. Small-study effects were assessed by cumulative forest plot [19, 20]. A sensitivity analysis was performed on high-quality studies as measured by the Newcastle-Ottawa scale. The statistical analysis was conducted using R studio v1.0.13 (The R Foundation for Statistical Computing) (package “meta v4.2”) [21].

## 3. Results

### 3.1. Study Selection and Study Characteristics

**Figure 1:**
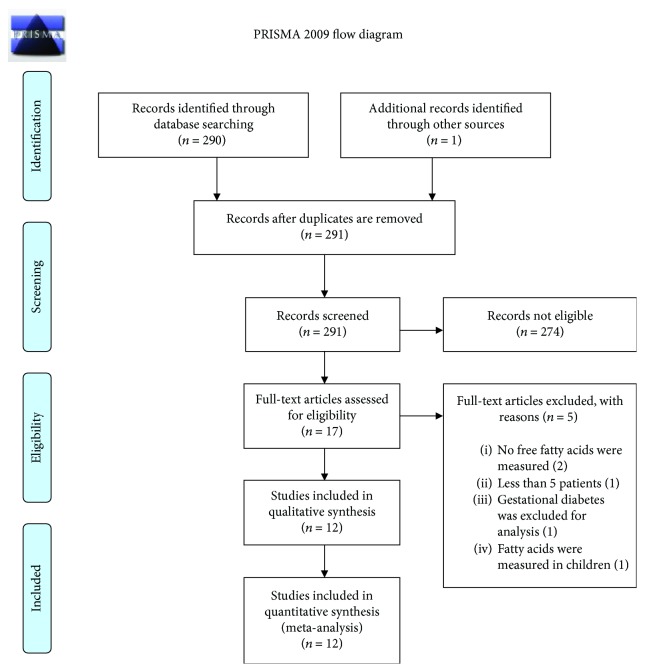


A total of 290 studies were identified by database searching, with one additional study included manually. Of them, 17 studies were eligible for full-text review. After review, twelve studies were retained for the systematic review and meta-analysis.  Figure 1 depicts the flow diagram according to the PRISMA recommendations.

From the five excluded studies, reasons for exclusion were as follows: two of them had no free fatty acid measurements [22, 23]; in a different study, free fatty acids were measured in neonates [24]. One study had less than five patients in one of the included arms [25], and the last study excluded women with gestational diabetes [26]. Characteristics of the included articles are listed in Table 1.

### 3.2. Risk of Bias of the Included Studies

Using the Newcastle-Ottawa scale for study quality assessment in observational studies, from a total of nine possible rating stars, only one study had six stars [27], mainly due to a lack of representativeness of the cases, no explicit selection of the controls, and lacking study controls for additional outcomes. Seven studies were awarded eight stars [28–34], all of them due to a lack of study controls for additional outcomes. The remaining four studies [35–38] were awarded nine stars. Supplemental [Supplementary-material supplementary-material-1] shows the full Newcastle-Ottawa scale assessment.

### 3.3. Synthesis of Results

A total of 2426 women were evaluated in the twelve included studies. From these, 21% (507/2426) had a diagnosis of having gestational diabetes. The mean gestational age at inclusion was 30.3 weeks of gestation (standard deviation (SD): 4); the majority of the included patients were in the second trimester of pregnancy, while the remaining 22% (527/2426) were in their third trimester. The maternal characteristics were the following: the mean maternal age was 29 years (SD 1.44), while the mean pregestational body mass index was 25 kg/m^2^ (SD 2.9) as measured in nine studies [28, 30–37]. The mean fasting glucose was 94.4 mg/dL (SD 22.7) as measured in seven studies [27, 30, 31, 34–37]. The mean fasting insulin was 12.66 *μ*IU/mL (SD 5.6) in seven studies [29–31, 34–37]. Only four studies [32, 33, 35, 38] measured Hb1Ac showing a mean of 4.9% (SD 0.5), while three studies [35, 37, 38] calculated the HOMA-IR, finding a mean index of 2.27 (SD 0.5).

### 3.4. Free Fatty Acids among Gestational Diabetes

**Figure 2:**
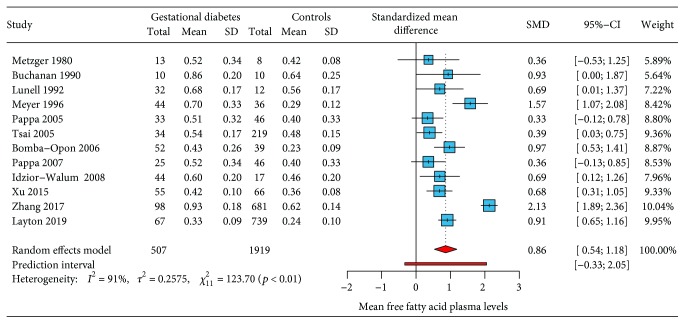


Twelve studies [27–38] had information about the mean levels of free fatty acids in gestational diabetes and controls. The standardized mean difference by random-effects modeling showed higher levels of free fatty acids among women with gestational diabetes (SMD: 0.86; 95% CI: 0.54-1.18; *p* < 0.001).  Figure 2 shows the forest plot with the individual results and the pooled estimates of free fatty acid plasma levels among gestational diabetes and controls.

**Figure 3:**
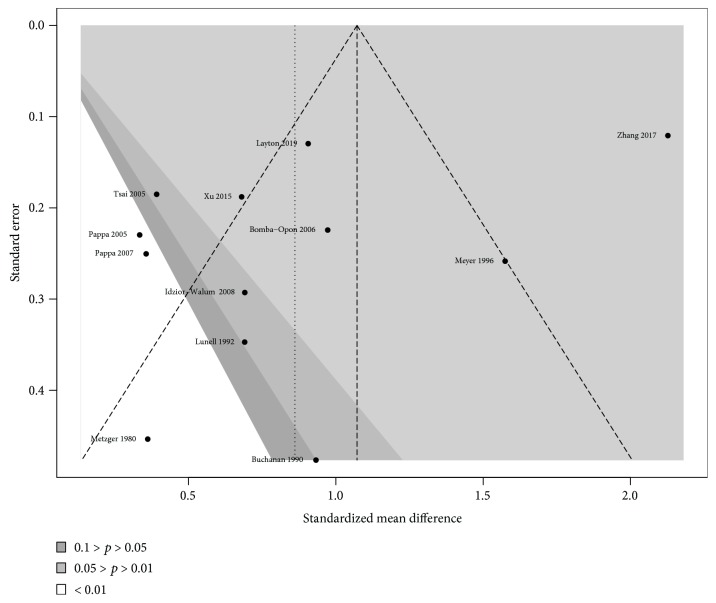


A *Q* value of 123.7 (*p* < 0.001) provides evidence that the effect size varies across studies. *I* ^2^ indicates that 91% of the depicted variation can be attributed to true effect rather than random error. A Baujat analysis showed that the majority of the heterogeneity comes from the study of Zhang (2017). Assessment of bias by contour-enhanced funnel plot depicted asymmetry of results when comparing the standardized mean difference to the standard error (size) of each study (Figure 3).

**Figure 4:**
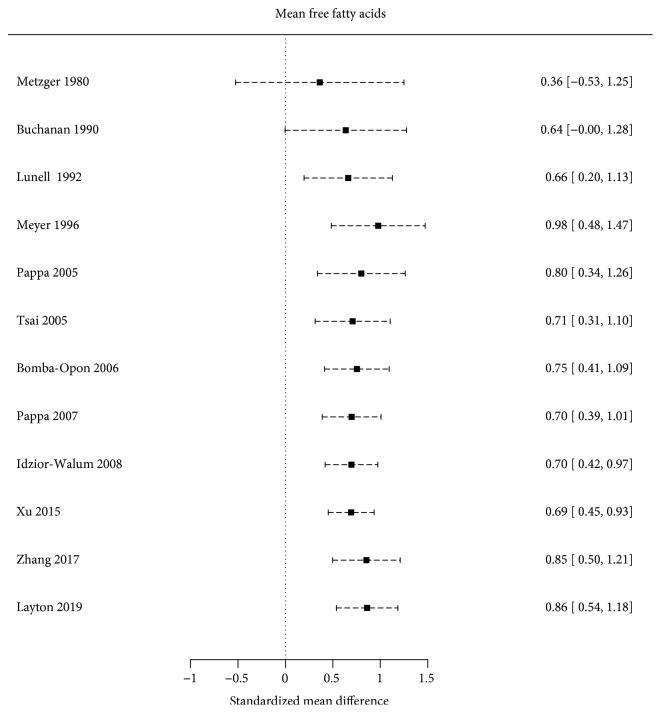


Nevertheless, when performing a linear regression to quantify the amount of heterogeneity, no evidence of bias was found by Egger's test (bias: -4.059; slope: 1.842; *p* = 0.111). Cumulative analysis depicted no trend towards greater effects in small studies (Figure 4).

A Copas selection model was performed to identify the probability of unpublished studies due to small effects in small studies, showing a 40% probability of unpublished studies due to this situation. Nonetheless, this model also showed that there is no unexplained study selection (*p* = 0.242), which reduces the likelihood of selection bias.

### 3.5. Subgroup Analysis of Measured Free Fatty Acid Plasma Levels according to the Trimester of Gestation

**Figure 5:**
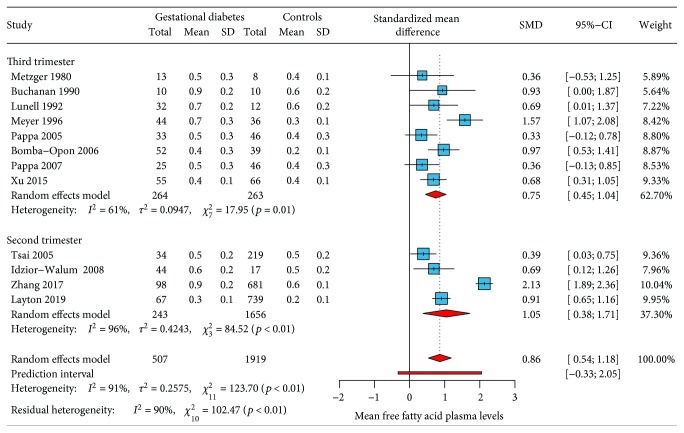


We performed a subgroup analysis to explain the heterogeneity found between studies and to compare the concentrations of free fatty acid plasma levels among trimesters of gestation. Pooled results showed higher plasma levels of free fatty acid among women with gestational diabetes mellitus during the second trimester of pregnancy (SMD: 1.05 vs. 0.75). Heterogeneity was partially explained by subgroup analysis, finding lower heterogeneity in studies measuring free fatty acid plasma levels in the third trimester rather than the second trimester (61% vs. 96%).  Figure 5 shows the subgroup analysis according to trimester of gestation.

### 3.6. Metaregression Analysis

**Figure 6:**
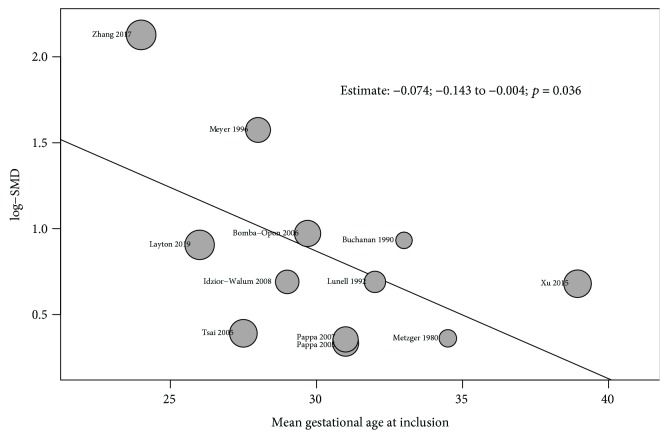


A metaregression was performed to identify the influence of several cofactors on the pooled result when at least five studies reported the needed information. From the evaluated variables, only the mean gestational age at inclusion was found to influence the result with a trend towards lower standardized mean difference of free fatty acid plasma levels at a later gestational age (estimate: -0.0741; 95% CI: -0.1436 to -0.0047; *p* = 0.036), explaining 31% of the heterogeneity found among results (Figure 6). For note, no significant changes were found on the plasma levels of FFA when comparing those studies quantifying FFA using a colorimetric procedure [30, 36] vs. those using enzyme immunoassay [28, 29, 31, 34, 35, 37, 38] vs. studies using both methods [32, 33] (QM = 4.224; *p* = 0.238).

Table 2 shows all calculations for the measured cofactors, the explainable contribution of heterogeneity that each one represents (*R* ^2^), and the residual heterogeneity (*I* ^2^).

## 4. Discussion

### 4.1. Main Findings

Free fatty acids have been proposed as a marker of insulin resistance in nonpregnant individuals. Higher plasma levels of these molecules have been found in type 2 diabetes and obesity. This study shows that plasma levels of free fatty acids are higher in women diagnosed with gestational diabetes mellitus (SMD: 0.86; 0.54-1.18; *p* < 0.001) and that this difference is higher during the second trimester of pregnancy (SMD: 1.05 vs. 0.75). The only extrinsic determinant influencing these results was the gestational age at which women were enrolled in each study, showing a significant decrease in the standardized mean difference of free fatty acid plasma levels as women were included at a later gestational age. No other cofactors such as pregestational BMI, fasting glucose, fasting insulin, or maternal age were found to influence the pooled results.

The reason for the elevation of free fatty acid plasma levels in gestational diabetes in comparison to normal pregnancies has not been well elucidated. The proposed underlying mechanism is a decrease in insulin secretion and insulin resistance in skeletal muscle induced by free fatty acids, resulting in an intramyocellular accumulation of diacylglycerol, which activates the protein kinase C cascade causing a reduction in the tyrosine phosphorylation of the insulin receptor substrate 1 (IRS-1). This pathway induces the activation of the PI3Kase, an important enzyme for insulin-stimulated glucose uptake [39]. Although similar abnormalities have been found in obese women with gestational diabetes [40, 41], no direct effects of FFA have been observed in the muscle of pregnant woman.

Another hypothesis could be that healthy pregnant women at 14-17 weeks of gestation, show an acute elevation of free fatty acids due to insulin resistance and a decrease in glucose oxidation in a dose-dependent manner [42]. Though we cannot know whether first trimester elevation of free fatty acids that contribute to the development of gestational diabetes or the production of hyperglycemic placental molecules such as tumor necrosis factor-*α* (TNF-*α*), resistin, and leptin induces an increase on free fatty acids [43], animal studies have shown that the intentional addition of free fatty acids in pregnant rabbits induce insulin resistance, while their reduction has the contrary effect [44, 45].

Differences of free fatty acids among trimester of gestation, being the lowest concentrations in the third trimester and the highest in the second trimester, could be explained by the pattern of insulin resistance that occurs during pregnancy. Insulin resistance reaches its peak during the second trimester (24-28 weeks' gestation) due to hormone-placental-related mechanism, including an increase in placental lactogen as the main contributor for insulin resistance [46]. The decrease of free fatty acid concentrations in the third trimester also reflects the diminished insulin resistance and the decrease in placental-related hormones responsible for this [11].

### 4.2. Clinical Implications

If free fatty acid plasma levels were to be elevated before pregnancy, during the first trimester and through the gestation, and if we could measure it in a longitudinal way in advance, the combination of a risk model that includes maternal characteristics plus biochemical parameters such as free fatty acid plasma levels, osteocalcin, fasting glucose, and fasting insulin could help improve the prediction of this disease earlier in pregnancy [14]. The importance of predicting a condition such as gestational diabetes in the first trimester is the possibility of an early intervention that could lead to a reduction in the incidence of diabetes later in gestation, along with the reduction in the number of adverse perinatal outcomes such as cesarean section and macrosomia.

### 4.3. Strengths and Limitations

There are several strengths in this analysis. Firstly, an exhaustive search and a blinded peer screening of all articles were performed to avoid potential biases. Secondly, the extraction of important cofactors that could be extrinsically or intrinsically affecting the results was performed, allowing us to produce several calculations such as subgroup analysis and metaregression to explain the possible heterogeneity and the influence that these cofactors have on the overall result. Finally, four types of test were performed to assess potential biases: funnel plot assessment to visually identify outliers, linear assessment for possible publication bias by Egger test, the probability of unpublished studies by Copas model, and the trend towards greater effect in small studies by cumulative forest plot.

But as there were several strengths, there were also weaknesses. The most important is the heterogeneity found among studies, which is very usual when performing standardized mean differences, telling us that results must be interpreted with caution. And the last weakness is the lack of studies measuring Hb1AC and HOMA-IR, which did not allow us to evaluate the influence that these variables had on the result.

### 4.4. Conclusions and Implications

Women with gestational diabetes have higher free fatty acid plasma levels when compared to healthy pregnant women. This difference is higher earlier in pregnancy, and as the gestation advances, the concentration of free fatty acids in diabetic women also declines. More research is needed to assess the performance of free fatty acids for the prediction of gestational diabetes during the first trimester of pregnancy.

## Figures and Tables

**Table 1:** Characteristics of included studies.

Author	Year	Country	Type of study	Inclusion criteria	Exclusion criteria	Diagnostic criteria for gestational diabetes	Gestational age at enrollment	Study population	Women with GDM	Mean pre-gestational BMI (Kg/m^2^)	Mean maternal age (years)	Mean fasting plasma glucose (mg/dL)	Mean fasting plasma insulin (uIU/mL)
Metzger	1980	USA	Cross-sectional	Women with gestational diabetes (fasting plasma glucose within the normal range for pregnancy or greater than 105 mg/dl) or normal carbohydrate metabolism.	None	One-step: 100g Fasting blood glucose ≥ 100 mg/dL; 1h ≥ 170 mg/dL; 2h ≥ 120 mg/dL; 3h ≥ 110mg/dL.	30-39 w	21	13	N/S	28.06	96	N/S

Buchanan	1990	USA	Nested case-control	Over-weight pregnant women (pre-pregnancy weight > 120% of ideal) without gestational diabetes mellitus or weight-match pregnant women with gestational diabetes.	Receiving exogenous insulin treatment before the study. Pre-pregnancy complications. Ingesting medications other than vitamins or iron.	Two-steps: First step; 50g. Second step: 100g cut point values, fasting 105 mg/dL; 1h: 190 mg/dL; 2h 165 mg/dL; 3h 145 mg/dL.	30-36 w	20	10	N/S	28.3	N/S	24.5

Lunell	1992	Sweden	Case-control	Pregnant women from the antenatal clinic in Kuwait City, where the indications for an oral glucose tolerance test were diabetic heredity of the first degree, the birth of a baby above 4500g or glucosuria	Known diabetic before their pregnancy. Taking medicines known to affect glucose tolerance.	Two-steps: First step; 50g. Second step: cut point values 2h: 5.8 mmol/l or 8.9 mmol/l over some other time point	30-34 w	44	32	31	N/S	78.55	13

Meyer	1996	New Zealand	Nested case-control	Women in the Illawarra area tested for GDM at the beginning of the third trimester	None	One-step: 75g; 2-hour: >8.0 mmol/l	Beginning of the third trimester	80	44	25.7	27	N/S	10.9

Pappa	2005	Greece	Case-control	Normal adult non-pregnant women, normal pregnant women with uncomplicated pregnancy or women with gestational diabetes mellitus.	Inborn errors of metabolism, epilepsy, chronic renal failure. Problems that could interfere with measurement.	Two-steps: First step; 50g. Second step: 100g cut point values, fasting 105 mg/dL; 1h: 190 mg/dL; 2h 165 mg/dL; 3h 145 mg/dL.	30-33 w	119	33	25.48	28	N/S	N/S
Tsai	2005	China	Nested case-control	Singleton pregnancies Positive diabetic screening test result at 24 to 31 weeks' gestation From Taipei's Municipal Women´s and Children´s Hospital and followed until delivery	Hypertension, hyperlipidemia, renal or liver disease, heart disease, thyroid disorder, pre-gestational diabetes mellitus, and multifetal pregnancy.	Two-steps: 50-g oral glucose; >7.8 mmol/l. Fasting 75g; >5.3 1h: 10.0 mmol/l; 2h: 8.6 mmol/l.	24-31w	253	219	22.3	31.6	86.48	10.5

Bomba-Opon	2006	Poland	Case-control	Pregnant women From Outpatient Clinic at the 1^st^ Department of Obstetrics and Gynecology Warsaw University School Medicine with or without gestational diabetes	None	Two-steps: 50 g if >139 mg/dL mmol/L, 75g. Cut values: Fasting>100 mg/dL, 1h >179 mg/dL, 3h >139 mg/dL	29.7w	91	52	24.4	28.8	N/S	N/S

Pappa	2007	Greece	Case-control	Pregnant women with uncomplicated pregnancy and women with gestational diabetes at 30-33 weeks of gestation Singleton pregnancy.	Inborn errors of metabolism, epilepsy, chronic renal failure. Problems that could interfere with measurements.	Two-steps: 50-g oral glucose; 1 h:> 130 mg / dl (7.2 mmol /l) Fasting 100g; 1h: 10.0mmol; 2h: 8.6mmol/l; 3h: 7.8mmol/l.	30-33w	71	25	25.4	27.8	N/S	N/S

Idzior-Walum	2008	Poland	Cross-sectional	Pregnant women referred to the outpatient diabetic clinic with suspicion of gestational diabetes	None	Two-steps: 50-g oral glucose; >7.8 mmol / l) Fasting 75g; >5.3 1h: 10.0mmol; 2h: 8.6mmol/l;	26-32w	61	44	27.8	28.3	82.87	13.5

Xu	2015	China	Case-control	Pregnant women with prenatal examinations and a cesarean section and delivered a single full-term healthy child.	Women with cardiovascular disease, endocrine disease, renal disease, hepatic disease, complications of gestation or any other disease conditions.	Fasting plasma glucose > 5.1 mmol/l and/or a 1-h glucose > 10.0 mmol/l and/or 2-h glucose	Before delivery	121	55	21.24	31.23	81.97	8.57

Zhang	2017	China	Cross-sectional	Chinese pregnant women with detailed visits for prenatal care in Hangzhou, China	Preexisting diabetes, overt thyroid disorder, endocrinopathies, renal insufficiency, corticosteroid therapy, or known fetal anomaly.	One-step: 75g Fasting blood glucose ≥ 5.1 mmol/l; 1h ≥ 10.0 mmol/l; 2h ≥ 8.5 mmol/l.	24-28 w	779	98	N/S	27.83	76.93	N/S

Layton	2019	Canada	Nested case-control	Pregnant women with data in the Genetics of Glucose regulation in Gestational and Growth (Gen3G) cohort. First trimester through delivery	Women with pre-existing diabetes at enrolment.	One-step: 75g Fasting blood glucose ≥ 92 mg/dL; 1h ≥ 180mg/dL; 2h ≥ 153 mg/dL.	Second trimester	805	67	24.16	28.08	N/S	7.63

N/S: not stated; USA: United States of America; w: weeks; h: hours; IU: international units; mg/dL: milligrams over deciliters; mmol/L: millimoles over liters.

**Table 2:** Metaregression analysis on the influence of several cofactors on the mean free fatty acid plasma levels.

Characteristic	Estimate	95% CI	*p* value	*I* ^2^%	*R* ^2^%	Number of studies
Gestational age at inclusion	-0.0741	-0.1436 to -0.0047	0.036	78	31	12
Pregestational BMI	0.0160	-0.0759 to 0.1078	0.733	61	3.4	9
Fasting glucose	0.0008	-0.0229 to 0.0245	0.947	88	0.1	7
Fasting insulin	0.0035	-0.0625 to 0.0695	0.917	58	0	7
Mean maternal age	-0.1542	-0.3765 to 0.0682	0.174	84	18	11
Year of publication	0.0143	-0.0152 to 0.0438	0.343	84	8.6	12

## Additional Points

*Condensation.* This meta-analysis provides evidence that plasma free fatty acids are higher in women with gestational diabetes mellitus when compared to healthy pregnant women.

## Supplementary Materials

Supplementary Materials Annex 1: search strategy and query syntax. Supplemental Table 1: Newcastle-Ottawa scale for case-control studies.

## References

[1] Kampmann U., Madsen L. R., Skajaa G. O., Iversen D. S., Moeller N., Ovesen P. (2015). Gestational diabetes: a clinical update. *World Journal of Diabetes*.

[2] Farrar D., Simmonds M., Bryant M. (2016). Hyperglycaemia and risk of adverse perinatal outcomes: systematic review and meta-analysis. *BMJ*.

[3] Gurr M. I., Harwood J. L., Frayn K. N. (2002). *Lipid Biochemistry. An Introduction*.

[4] Sears B., Perry M. (2015). The role of fatty acids in insulin resistance. *Lipids in Health and Disease*.

[5] Roden M., Price T. B., Perseghin G. (1996). Mechanism of free fatty acid-induced insulin resistance in humans. *The Journal of Clinical Investigation*.

[6] Jensen M. D., Haymond M. W., Rizza R. A., Cryer P. E., Miles J. M. (1989). Influence of body fat distribution on free fatty acid metabolism in obesity. *The Journal of Clinical Investigation*.

[7] Boden G., Chen X., Ruiz J., White J. V., Rossetti L. (1994). Mechanisms of fatty acid-induced inhibition of glucose uptake. *The Journal of Clinical Investigation*.

[8] Boden G., Cheung P., Stein T. P., Kresge K., Mozzoli M. (2002). FFA cause hepatic insulin resistance by inhibiting insulin suppression of glycogenolysis. *American Journal of Physiology-Endocrinology and Metabolism*.

[9] Boden G. (1999). Free fatty acids, insulin resistance, and type 2 diabetes mellitus. *Proceedings of the Association of American Physicians*.

[10] Boden G. (2006). Obesity, insulin resistance, type 2 diabetes and free fatty acids. *Expert Review of Endocrinology and Metabolism*.

[11] Catalano P. M., Kirwan J. P., Haugel-de Mouzon S., King J. (2003). Gestational diabetes and insulin resistance: role in short- and long-term implications for mother and fetus. *The Journal of Nutrition*.

[12] Stroup D. F., Berlin J. A., Morton S. C. (2000). Meta-analysis of observational studies in epidemiology: a proposal for reporting. Meta-analysis of observational studies in epidemiology (MOOSE) group. *JAMA*.

[13] Moher D., Liberati A., Tetzlaff J., Altman D. G., PRISMA Group (2009). Preferred reporting items for systematic reviews and meta-analyses: the PRISMA statement. *Journal of Clinical Epidemiology*.

[14] Martinez-Portilla R. J., Villafan-Bernal J. R., Lip-Sosa D. L. (2018). Osteocalcin serum levels in gestational diabetes mellitus and their intrinsic and extrinsic determinants: systematic review and meta-analysis. *Journal Diabetes Research*.

[15] Wells G. A., Shea B., O'Connell D. The Newcastle-Ottawa scale (NOS) for assessing the quality of nonrandomised studies in meta-analyses. http://www.ohri.ca/programs/clinical_epidemiology/oxford.asp.

[16] White I. R., Thomas J. (2005). Standardized mean differences in individually-randomized and cluster-randomized trials, with applications to meta-analysis. *Clinical Trials*.

[17] Collab C. *Cochrane Handbook for Systematic Reviews of Interventions. 9.2.3.2 The standardized mean difference*.

[18] Borenstein M., Higgins J. P. T., Hedges L. V., Rothstein H. R. (2017). Basics of meta-analysis: *I*
^2^ is not an absolute measure of heterogeneity. *Research Synthesis Methods*.

[19] Lau J., Schmid C. H., Chalmers T. C. (1995). Cumulative meta-analysis of clinical trials builds evidence for exemplary medical care. *Journal of Clinical Epidemiology*.

[20] Borenstein M., Hedges L. V., Higgins J. P. T., Rothstein H. R. (2009). *Introduction to Meta-Analysis*.

[21] Schwarzer G. (2007). Meta: an R package for meta-analysis. *R News*.

[22] Hou W., Meng X., Zhao A. (2018). Development of multimarker diagnostic models from metabolomics analysis for gestational diabetes mellitus (GDM). *Molecular & Cellular Proteomics*.

[23] Nord E., Hanson U., Persson B. (1997). Assessment of therapy in gestational diabetes by substrate and hormone responses to a standardized test meal. *Diabetic Medicine*.

[24] Ortega-Senovilla H., Schaefer-Graf U., Meitzner K., Abou-Dakn M., Herrera E. (2013). Decreased concentrations of the lipoprotein lipase inhibitor angiopoietin-like protein 4 and increased serum triacylglycerol are associated with increased neonatal fat mass in pregnant women with gestational diabetes mellitus. *The Journal of Clinical Endocrinology and Metabolism*.

[25] Catalano P. M., Nizielski S. E., Shao J., Preston L., Qiao L., Friedman J. E. (2002). Downregulated IRS-1 and PPAR*γ* in obese women with gestational diabetes: relationship to FFA during pregnancy. *American Journal of Physiology-Endocrinology and Metabolism*.

[26] Chen X., Scholl T. O. (2008). Association of elevated free fatty acids during late pregnancy with preterm delivery. *Obstetrics and Gynecology*.

[27] Metzger B. E., Phelps R. L., Freinkel N., Navickas I. A. (1980). Effects of gestational diabetes on diurnal profiles of plasma glucose, lipids, and individual amino acids. *Diabetes Care*.

[28] Bomba-Opon D., Wielgos M., Szymanska M., Bablok L. (2006). Effects of free fatty acids on the course of gestational diabetes mellitus. *Neuro Endocrinology Letters*.

[29] Buchanan T. A., Metzger B. E., Freinkel N. (1990). Accelerated starvation in late pregnancy: a comparison between obese women with and without gestational diabetes mellitus. *American Journal of Obstetrics and Gynecology*.

[30] Layton J., Powe C., Allard C. (2019). Maternal lipid profile differs by gestational diabetes physiologic subtype. *Metabolism*.

[31] Meyer B., Calvert D., Moses R. (1996). Free fatty acids and gestational diabetes mellitus. *The Australian & New Zealand Journal of Obstetrics & Gynaecology*.

[32] Pappa K. I., Anagnou N. P., Salamalekis E. (2005). Gestational diabetes exhibits lack of carnitine deficiency despite relatively low carnitine levels and alterations in ketogenesis. *The Journal of Maternal-Fetal & Neonatal Medicine*.

[33] Pappa K. I., Vlachos G., Theodora M., Roubelaki M., Angelidou K., Antsaklis A. (2007). Intermediate metabolism in association with the amino acid profile during the third trimester of normal pregnancy and diet-controlled gestational diabetes. *American Journal of Obstetrics and Gynecology*.

[34] Tsai P. J., Yu C. H., Hsu S. P. (2005). Maternal plasma adiponectin concentrations at 24 to 31 weeks of gestation: negative association with gestational diabetes mellitus. *Nutrition*.

[35] Idzior-Walus B., Cyganek K., Sztefko K. (2008). Total plasma homocysteine correlates in women with gestational diabetes. *Archives of Gynecology and Obstetrics*.

[36] Lunell N. O., Persson B., Viktorin L. (1992). Urinary C-peptide in gestational diabetes. *South African Medical Journal*.

[37] Xu M., Liu B., Wu M. F., Chen H. T., Cianflone K., Wang Z. L. (2015). Relationships among acylation-stimulating protein, insulin resistance, lipometabolism, and fetal growth in gestational diabetes mellitus women. *Journal of Obstetrics and Gynaecology*.

[38] Zhang J., Yao J., Zhao Y., Zheng N., Dai Y. (2017). Association between serum free fatty acids levels and gestational diabetes mellitus: a cross-sectional study. *Clinical Laboratory*.

[39] Sivan E., Boden G. (2003). Free fatty acids, insulin resistance, and pregnancy. *Current Diabetes Reports*.

[40] Friedman J. E., Ishizuka T., Shao J., Huston L., Highman T., Catalano P. (1999). Impaired glucose transport and insulin receptor tyrosine phosphorylation in skeletal muscle from obese women with gestational diabetes. *Diabetes*.

[41] Garvey W. T., Maianu L., Hancock J. A., Golichowski A. M., Baron A. (1992). Gene expression of GLUT4 in skeletal muscle from insulin-resistant patients with obesity, IGT, GDM, and NIDDM. *Diabetes*.

[42] Sivan E., Homko C. J., Whittaker P. G., Reece E. A., Chen X., Boden G. (1998). Free fatty acids and insulin resistance during pregnancy. *The Journal of Clinical Endocrinology and Metabolism*.

[43] Desoye G., Hauguel-de Mouzon S. (2007). The human placenta in gestational diabetes mellitus. The insulin and cytokine network. *Diabetes Care*.

[44] Hauguel S., Gilbert M., Girard J. (1987). Pregnancy-induced insulin resistance in liver and skeletal muscles of the conscious rabbit. *The American Journal of Physiology*.

[45] Gilbert M., Basile S., Baudelin A., Pere M. C. (1993). Lowering plasma free fatty acid levels improves insulin action in conscious pregnant rabbits. *American Journal of Physiology-Endocrinology and Metabolism*.

[46] Barbour L. A., McCurdy C. E., Hernandez T. L., Kirwan J. P., Catalano P. M., Friedman J. E. (2007). Cellular mechanisms for insulin resistance in normal pregnancy and gestational diabetes. *Diabetes Care*.

