# Supplementary material for: Plasma Levels of Free Fatty Acids in Women with Gestational Diabetes and Its Intrinsic and Extrinsic Determinants: Systematic Review and Meta-Analysis
Source: J Diabetes Res. 2019 Aug 18;2019:7098470. doi: 10.1155/2019/7098470 (PMC6721400; doi:10.1155/2019/7098470)
Supplement: Supplementary Materials — Annex 1: search strategy and query syntax. Supplemental Table 1: Newcastle-Ottawa scale for case-control studies. [file 7098470.f1.pdf]

Supplemental table 1. Newcastle-Ottawa scale for case-control studies

Study		Selection				Comparability		Exposure			Stars
Author	Year	Is the case definition adequate?	Representativeness of the cases	Selection of controls	Definition of controls	Study controls for the main outcome	Study controls for additional outcomes	Ascertainment of exposure	Same method of ascertainment for cases and controls	Non-Response rate	
Lunell	1992	*	*	*	*	*	*	*	*	*	9
Meyer	1996	*	*	*	*	*	-	*	*	*	8
Tsai	2005	*	*	*	*	*	-	*	*	*	8
Bomba Opon	2006	*	*	*	*	*	-	*	*	*	8
Pappa	2007	*	*	*	*	*	-	*	*	*	8
Idzior-Walus	2008	*	*	*	*	*	*	*	*	*	9
Buchanan	1990	*	*	*	*	*	-	*	*	*	8
Layton	2018	*	*	*	*	*	-	*	*	*	8
Pappa	2005	*	*	*	*	*	-	*	*	*	8
Xu	2015	*	*	*	*	*	*	*	*	*	9
Zhang	2017	*	*	*	*	*	*	*	*	*	9
Metzger	1980	*	-	-	*	*	-	*	*	*	6

PUBMED: 112

("fatty acids, nonesterified"[MeSH Terms] OR ("fatty"[All Fields] AND "acids"[All Fields] AND "nonesterified"[All Fields]) OR "nonesterified fatty acids"[All Fields] OR ("free"[All Fields] AND "fatty"[All Fields] AND "acids"[All Fields]) OR "free fatty acids"[All Fields]) AND ("diabetes, gestational"[MeSH Terms] OR ("diabetes"[All Fields] AND "gestational"[All Fields]) OR "gestational diabetes"[All Fields] OR ("gestational"[All Fields] AND "diabetes"[All Fields])) AND "humans"[MeSH Terms]

SCOPUS: 123

TITTLE-ABS-KEY (free AND fatty AND acids AND gestational AND diabetes)

WOS: 55

TEMA: (free fatty acids) *AND***TEMA:** (gestational diabetes)

Refinado por: [excluyendo] **Bases de datos:** (MEDLINE)

Período de tiempo: Todos los años.**Bases de datos:** WOS, BIOSIS, CABI, CCC, DIIDW, KJD, MEDLINE, RSCI, SCIELO, ZOOREC.

Idioma de búsqueda=Auto
